# Replica Exchange Molecular Dynamics of Diphenylalanine Amyloid Peptides in Electric Fields

**DOI:** 10.1021/acs.jpcb.1c01939

**Published:** 2021-05-14

**Authors:** Brajesh Narayan, Colm Herbert, Brian J. Rodriguez, Bernard R. Brooks, Nicolae-Viorel Buchete

**Affiliations:** † School of Physics, University College Dublin, Belfield, Dublin 4, Ireland; ‡ Institute for Discovery, University College Dublin, Belfield, Dublin 4, Ireland; § Conway Institute of Biomolecular and Biomedical Research, University College Dublin, Belfield, Dublin 4, Ireland; ∥ Laboratory of Computational Biology, NHLBI, National Institutes of Health, Bethesda, Maryland 20892, United States

## Abstract

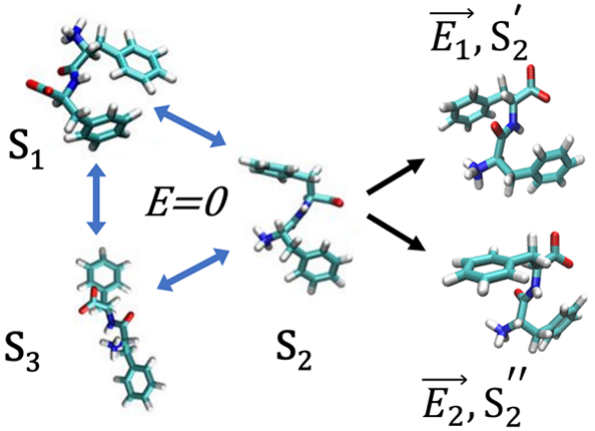

The self-assembling propensity of amyloid peptides such as diphenylalanine (FF) allows them to form ordered, nanoscale structures, with biocompatible properties important for biomedical applications. Moreover, piezoelectric properties allow FF molecules and their aggregates (e.g., FF nanotubes) to be aligned in a controlled way by the application of external electric fields. However, while the behavior of FF nanostructures emerges from the biophysical properties of the monomers, the detailed responses of individual peptides to both temperature and electric fields are not fully understood. Here, we study the temperature-dependent conformational dynamics of FF peptides solvated in explicit water molecules, an environment relevant to biomedical applications, by using an enhanced sampling method, replica exchange molecular dynamics (REMD), in conjunction with applied electric fields. Our simulations highlight and overcome possible artifacts that may occur during the setup of REMD simulations of explicitly solvated peptides in the presence of external electric fields, a problem particularly important in the case of short peptides such as FF. The presence of the external fields could overstabilize certain conformational states in one or more REMD replicas, leading to distortions of the underlying potential energy distributions observed at each temperature. This can be overcome by correcting the REMD initial conditions to include the lower-energy conformations induced by the external field. We show that the converged REMD data can be analyzed using a Markovian description of conformational states and show that a rather complex, 3-state, temperature-dependent conformational dynamics in the absence of electric fields collapses to only one of these states in the presence of the electric fields. These details on the temperature- and electric-field-dependent thermodynamic and kinetic properties of small FF amyloid peptides can be useful in understanding and devising new methods to control their aggregation-prone biophysical properties and, possibly, the structural and biophysical properties of FF molecular nanostructures.

## Introduction

Small, biocompatible peptides, such as amyloid-forming diphenylalanine (FF), have raised an increasing interest in both theoretical^1−5^ and experimental^6−10^ nanoscience studies for almost two decades. This success is due both to their intrinsic propensity to self-assemble in a hierarchic manner from FF monomers into diverse nanostructures and to the interesting emerging biophysical properties of these nanostructures (e.g., piezoelectric, optical, and mechanical strength properties).^7,8,11^ FF is one of the smallest, naturally occurring amyloid peptides, found commonly in the hydrophobic structural core of the amyloid beta (Aβ) protein, which allowed its identification as one of the smallest peptides capable of self-assembly leading to the formation of ordered fibrillar amyloid nanostructures.^11^

Amyloid FF peptides and their bioinspired nanoscale structures such as FF and nanotubes, nanospheres, or even nanorods^12^ have led to a multitude of applications in biomedicine, nanoscience, and nanotechnology.^5,7,8,13^ However, there are also significant limitations to using FF-based nanomaterials, one of the main factors being the instability of FF nanotubes in solution (e.g., a major limitation hindering the development of FF nanotube-based biosensors or drug delivery systems) and the relative heterogeneity of the local, nm-scale structures formed by self-assembly of the FF peptides under various conditions, including but not limited to temperature, pH, and solvation.^9,10^ To overcome such barriers it becomes important to understand and control the peptide self-assembly process. Innovative approaches such as directed self-assembly have been developed, such as subjecting a system to the influence of externally applied stimuli, including mechanical mixing, temperature, or pH variations. Thus, different degrees of control are achieved by enabling the tuning of desired interactions, structure, and properties of the final self-assembled nanomaterials. Recent experimental methods of directed self-assembly, such as dielectrophoresis, rely on applying an external electric field on the entire ensemble of assembling peptides and have been used to modulate the alignment of FF nanotubes.^14−18^ However, a main challenge with directed self-assembly remains the need for predictive models that bridge the detailed conformational behavior of a single FF molecule under an electric field and the properties of the resulting nanoscale self-assembled structures.

In this study, we use atomistic molecular dynamics (MD) simulations to study the combined effect of applied electric fields and temperature dependence on the detailed conformational dynamics of FF peptides solvated in explicit water molecules, an environment relevant to biomedical applications. In order to capture the temperature effect on the FF thermodynamics and kinetic properties, our simulations rely on an enhanced sampling method, temperature replica exchange molecular dynamics (REMD). Here, we first highlight and overcome a possible problem that may lead to artifacts during the setup of REMD simulations of explicitly solvated peptides in the presence of external electric fields, a problem particularly important in the case of short peptides such as FF. We show how to overcome this problem (i.e., by correcting the REMD initial conditions to include the lower-energy conformations induced by the external field), and we analyze the converged REMD data using a Markovian description of conformational states of the simulated system. Finally, we discuss the observed temperature- and electric-field-dependent thermodynamic and kinetic properties of small FF amyloid peptides, which may be useful in understanding and devising new methods to control their aggregation-prone biophysical properties and, possibly, the structural and biophysical properties of FF molecular nanostructures.

## Methods

### REMD Simulations in External Electric Fields

We use atomistic REMD simulations of FF peptides, following a similar procedure to our previous study described in ref (4) (though, in that case we did not use external electric fields), with the MD package Gromacs (version 5.1.4),^19,20^ using Langevin dynamics with a friction coefficient of 0.1 ps^–1^.^21^ These REMD simulations used the particle-mesh Ewald implementation with a switching distance for the van der Waals interactions and nonbonded electrostatics of 8.5 Å and a cutoff distance of 12 Å, and an integration time step of 2 fs. The runs were performed in the NPT ensemble, using an improved Berendsen-type weak coupling method for temperature coupling,^22^ Parrinello–Rahman isotropic pressure coupling,^23^ the recent CHARMM^24^ 36 all-atom protein force field parameters (C36),^25^ and explicit TIP3P^26^ water molecules. The FF peptide was included in a simulation box containing 1112 water molecules. To enhance the sampling, REMD is performed with 12 replicas running in parallel at temperature values chosen according to an optimized protocol^27^ (Table 1) in the range 310.00–373.45 K.^28^

**Table 1:** Three Main Sets of REMD Data Analyzed Here Correspond to Simulations, with Explicit Water Molecules, Performed in the Presence of External Electric Fields with Intensities of (a) 0, (b) 30, and (c) 45 kcal/(mol Å e), Separately^a^

(a) *E* = 0 kcal/(mol Å e)												
replica no.	1	2	3	4	5	6	7	8	9	10	11	12
temp [K]	310.00	315.38	320.82	326.35	331.96	337.64	343.41	349.26	355.19	361.20	367.30	373.45
time [ns]	126	126	126	126	126	126	126	126	126	126	126	126
(b) *E* = 30 kcal/(mol Å e)												
replica no.	1	2	3	4	5	6	7	8	9	10	11	12
temp [K]	310.00	315.38	320.82	326.35	331.96	337.64	343.41	349.26	355.19	361.20	367.30	373.45
time [ns]	100	100	100	100	100	100	100	100	100	100	100	100
(c) *E* = 45 kcal/(mol Å e)												
replica no.	1	2	3	4	5	6	7	8	9	10	11	12
temp [K]	310.00	315.38	320.82	326.35	331.96	337.64	343.41	349.26	355.19	361.20	367.30	373.45
time [ns]	98	98	98	98	98	98	98	98	98	98	98	98

a Each run used 12 replicas, at temperatures spaced according to an optimized protocol,^27^ as indicated in the table together with the corresponding run times. The total simulation time is ∼4 μs [i.e., including also the initial setup and testing runs at 30 kcal/(mol Å e)].

Table 1 includes details on the main REMD simulation runs analyzed here, together with the corresponding temperature values. The three REMD data sets were generated in the presence of external electric fields with intensities of (a) 0, (b) 30, and (c) 45 kcal/(mol Å e), respectively. Overall, the total simulation time necessary was more than 4 μs of REMD dynamics [i.e., including also the initial setup and testing runs at 30 kcal/(mol Å e)].

For the REMD simulations, we prepared the system including the FF amyloid peptide and water molecules using VMD’s^29^ Molefacture Plugin protein builder tool, followed by minimization, heating, and equilibration stages, at each electric field value. The system was simulated using the Gromacs REMD implementation,^27^ with an average acceptance probability for the replica exchanges of ∼20%. The atomic velocities and coordinates were saved every 100 fs, and after the simulation, the REMD per-replica trajectory data (i.e., referred to as R-trajectories) were also transformed for analysis into per-temperature data (i.e., referred to as T-trajectories) using the Gormacs *demux* command. The Gromacs *trajconv* command was used to select system conformations every 1 ps (i.e., every 500th MD frame, with a 2 fs integration time step) for our detailed thermodynamic and kinetic analysis.

For the first run, in the absence of electric fields, the production simulations were done for 126 ns for each of the 12 replicas, giving a total REMD simulation time of 1.512 μs, which was sufficient for achieving convergence of all the relevant thermodynamic and kinetic quantities. As an additional test for convergence, we also checked the “equal occupancy rule” of replicas at each temperature,^30^ which is a very useful method for assessing quickly the performance of parallel tempering simulations.^30,31^ Subsequently, kinetic data on the identified conformational Markov states and the corresponding transition probabilities were calculated from the REMD trajectories as discussed below.

### Extracting Transition Probabilities and Rates from REMD Data

To identify and test the Markovian conformational states and the corresponding transition states for REMD trajectories, we analyzed the temperature-dependent FF data by following the workflow that we developed and presented in our previous study,^4^ and the corresponding transition probabilities were extracted and compared. As highlighted in ref (28), the replica R-trajectories are continuous, even though they travel at various temperatures during the REMD as exchange attempts are accepted (e.g., Figure 1 in ref (28)), while the data captured as T-trajectories are actually discontinuous, being interrupted at time steps when exchange attempts are accepted. Note that, unlike other REMD analysis methods that are focused on T-trajectories, due to their well-defined temperatures, we showed that it is convenient to start by analyzing R-trajectories in order to take advantage of their time-continuity both in the initial assignment of states and, importantly, in assessing convergence.^28,32^ As demonstrated in ref (28), there is an analytical relation that connects both R-trajectories and T-trajectories. The propagators (i.e., conditional probabilities) for transitions along R-trajectories were shown to be in effect the weighted geometric means of propagators extracted for the corresponding transitions in T-trajectories. This observation enables a powerful direct application of kinetic analysis along R-trajectories, on which state assignment is easier due to their continuous nature, rather than performing directly a more laborious (and thus more prone to errors) kinetic analysis of the discontinuous T-trajectories.^28,32^

Following the procedure detailed for REMD data of FF peptides in ref (32), here we assume that the conformational space of a system can be discretized into *N* distinct states that obey a master equation, which can be expressed in matrix notation as , with **p**(*t*) being the time-dependent column vector of probabilities with elements such that *p*_*n*_(*t*) > 0, *n* ∈ {1, ..., *N*}. Here, **K**(*t*) is the *N* × *N* rate matrix, the **K** element *k*_*nm*_ is the rate of transition from state *m* to state *n*, and *p*_*m*_ is the probability of the state labeled *m* at time *t*.^33−44^ At thermodynamic and kinetic equilibrium, we have **Kp°** ≡ 0, with **p°** being thus the vector of equilibrium populations that has positive elements, *p*_*n*_° > 0, *n* ∈ {1, ..., *N*}, and it is properly normalized (). Therefore, **p°** appears as the first right eigenvector of **K**, corresponding to the first eigenvalue λ_1_ = 0.

**Figure 1:**
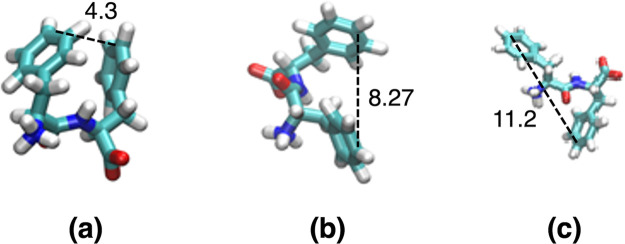
Representative conformations of FF peptides in the absence of externally applied electric fields. Values of the *d*_ee_ distances (i.e., distances between the C_*ζ*_ atoms at the ends of the two side chains, in Å) are shown in black.

Similarly to previous studies,^4,32^ we use the Markov-based direct transition counting (DTC) method^32,45^ for extracting transition rates from REMD trajectories (in this case, for different values of an externally applied electric field), which requires the initial assignment of conformational states of the system. The conformational states of the peptide are assigned by following each replica using both T-trajectories and R-trajectories, using the transition based assignment (TBA) method described and used in previous studies.^28,41,46^ We use the TBA method of assignment of Markov states for biomolecular MD trajectories introduced in ref (41) and reviewed in detail subsequently in ref (46). The TBA method requires initially a reasonable choice of reaction coordinates that allow a good discrimination between the different conformational Markov states. However, though these reaction coordinates need to be reasonably good, the subsequent state assignment step does not depend entirely on their absolute quality, as the TBA method also uses additional, more specific information from analyzing the actual transition paths (i.e., time sequence of transition events) to the state assignment process.^46^ As described next here, we use the *d*_ee_ distances (i.e., distances between the C_*ζ*_ atoms at the ends of the two side chains, in Å), illustrated in Figure 1 in black, as a useful choice for initiating the TBA analysis step.

### Tests of REMD Convergence

Following previous studies,^4^ we tested initially the REMD data convergence by investigating the “equal occupancy” rule of replicas at each temperature,^30^ which is fast and useful to assess the performance of parallel tempering simulations.^30,31^ Additionally, we have also analyzed and compared data from both R- and T-trajectories to show that our extracted quantities are converged (e.g., as shown in the probability distributions of different relevant observables illustrated in Figures 5–8). As discussed in ref (4), it is important to note that, in cases with several Markovian states present (here, for the FF dynamics in the absence of electric fields), the transition probabilities extracted from REMD data after applying the TBA method to project the R- and T-trajectories to states and performing the kinetic analysis can also serve as the “ultimate” test of the convergence of the REMD simulations performed. In practice, we can also use blocks of REMD data to estimate statistical errors for the extracted transition probabilities, as errors of the means for each data block. The analysis of the errors in the extracted intrinsic parameters of the Markovian kinetics offers a reliable assessment of the convergence of the data in the MD trajectories generated.^4^

## Results and Discussion

We generate and use new data from REMD simulations performed in the presence of external electric fields to probe the combined T-field- and E-field-dependent conformational dynamics of FF peptides (Figure 1).^3,4^ However, while the REMD simulations without electric fields were rather straightforward, the presence of the external field allowed us to unveil interesting artifacts. These may occur in general during the setup of any REMD simulations of explicitly solvated peptides in the presence of external electric fields, though they have a particularly high likelihood in the case of short peptides such as FF. In this case, the presence of the external fields can induce rapidly (i.e., on the order of 10s of picoseconds) and overstabilize (i.e., as compared to conformational dynamics in the absence of external fields) a low-energy conformational state in one or more REMD replicas, leading to distortions of the underlying potential energy distributions observed at each temperature.

**Figure 2:**
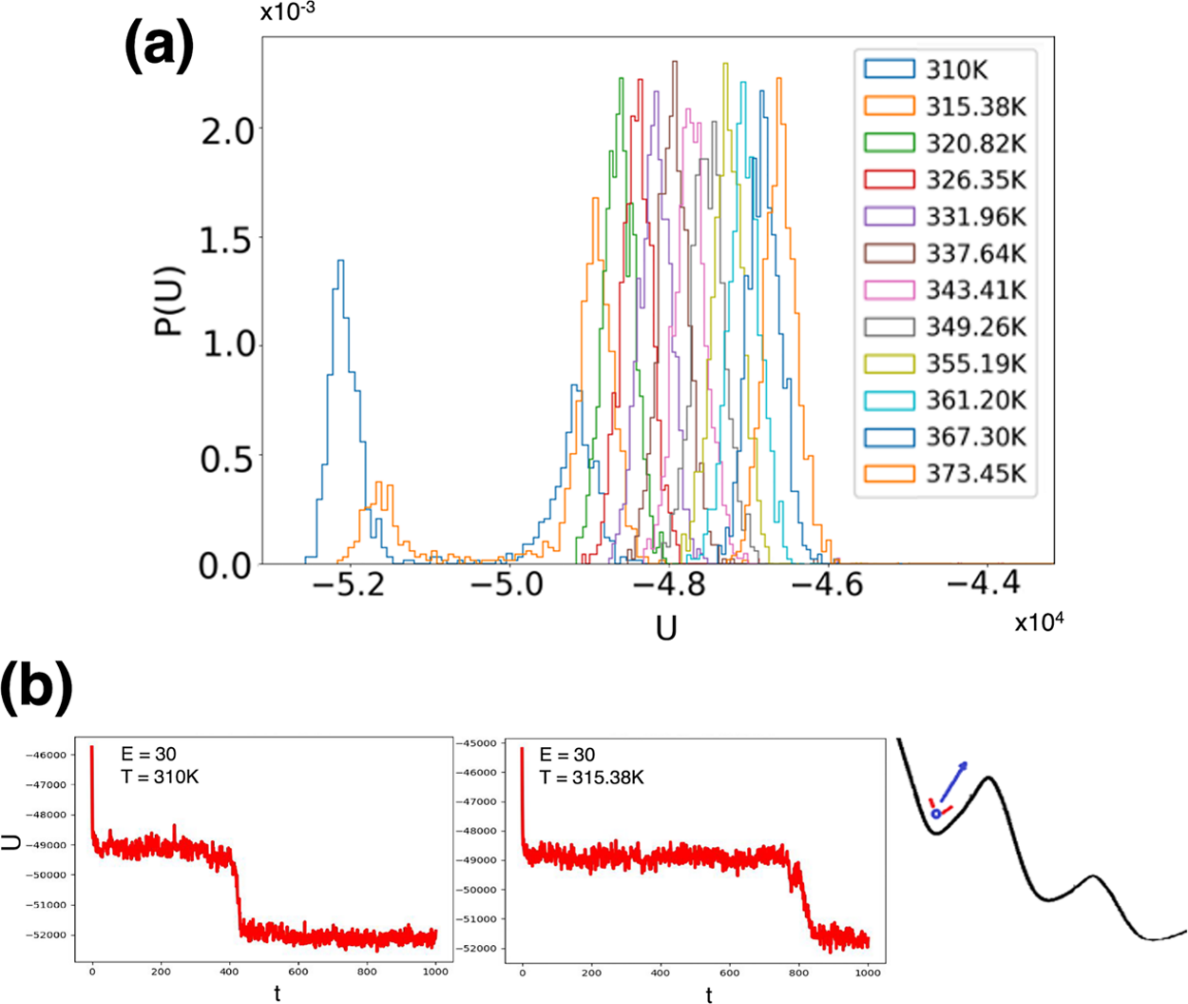
(a) Distributions of potential energy values (*U*, in kcal/mol) calculated from REMD simulations in the presence of an external electric field with an intensity of *E* = 30 kcal/(mol Å e). (b) Illustration of the problems that could occur when attempting REMD simulations in external electric fields. The presence of the field can induce some (in this case the first two) replicas to adopt conformations that are significantly lower in energy than the corresponding initial conformational states of the other replicas. This is a serious artifact, as illustrated in part a, as it changes the expected equilibrium *U* distributions.

The issue is illustrated in Figure 2 that shows the potential energy distributions for our initial replica exchange FF simulation with explicit water molecules in an electric field of intensity *E* = 30 kcal/(mol Å e). The non-Gaussian shape of the potential energy distribution is evidenced for the first two lowest temperatures as shown in Figure 2a, while the induced transitions to field-stabilized low-energy conformations are illustrated schematically in Figure 2b. The REMD implementation in most software packages, and the underlying replica exchange attempts, is designed to preserve a detailed balance when sampling from canonical distributions. Thus, the REMD exchange protocol relies on accurate dynamics that preserves the Gaussian shape of the underlying potential energy distributions. Parameters of the REMD simulations such as the number of replicas and the exact values of the temperatures selected depend directly on the correct shape of the underlying energy distributions and on their overlap (e.g., which controls the acceptance/rejection exchange probabilities for a simulation of a system with a certain number of atoms and the corresponding thermodynamic conditions). Thus, replica potential energy distributions with non-Gaussian shapes due to, in this case, the presence of external fields can easily lead to serious artifacts. We note that this cause is different from REMD artifacts due to modified underlying energy distributions due, for example, to the use of week-coupling thermostats which were highlighted before.^47^ Earlier studies have shown that, in REMD simulations of other small peptides, such as dialanine^47^ and pentaalanine^48^ using explicit TIP3P water molecules,^26^ the choice of weak-coupling thermostats can significantly affect the outcome of REMD simulations, though in that case through a narrowing of the underlying potential energy distribution for each replica.

**Figure 3:**
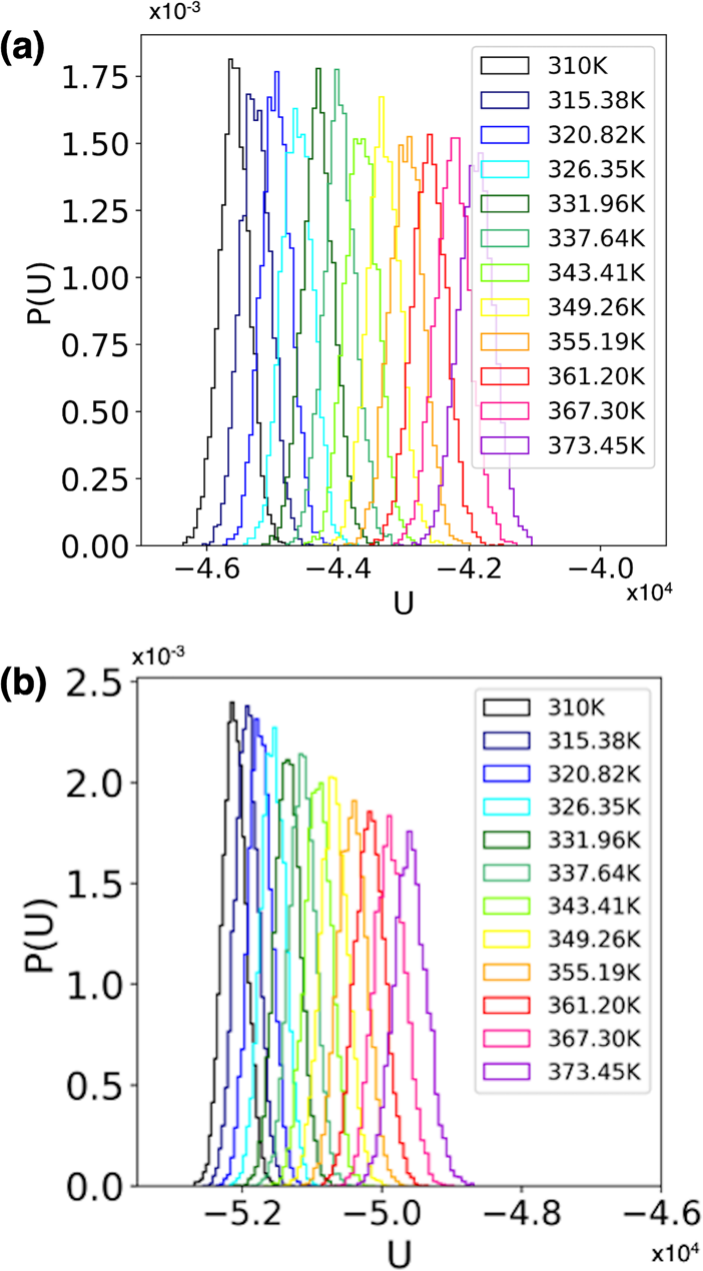
Distributions of potential energy values (*U*, in kcal/mol) calculated from REMD simulations (a) at *E* = 0 kcal/(mol Å e) and (b) with corrected initial conditions in the presence of an external electric field with an intensity of *E* = 30 kcal/(mol Å e).

The artifacts due to external electric fields can be overcome, as demonstrated here, by correcting the REMD initial conditions to include the lower-energy conformations induced by the external field for all replicas. When transitioning from properly equilibrated initial conditions to simulations when an additional field is present, it is thus crucial to not only re-equilibrate but also monitor the underlying energy distributions for all replicas (see Figure 3), at all temperatures, and reinitialize the REMD protocol to include the lower-energy conformations that may be induced. Subsequently, the REMD protocol can proceed to achieve enhanced sampling by use of replicas running at higher temperatures, in parallel, while preserving the correct underlying dynamic and thermodynamic behavior of the system at all temperatures. Figure 3a shows the corrected REMD distributions of potential energy values (*U*, in kcal/mol) calculated from REMD simulations at *E* = 0 kcal/(mol Å e) (Figure 3a), and also with the new, corrected initial conditions in the presence of an external electric field with an intensity of *E* = 30 kcal/(mol Å e) (Figure 3b).

**Figure 4:**
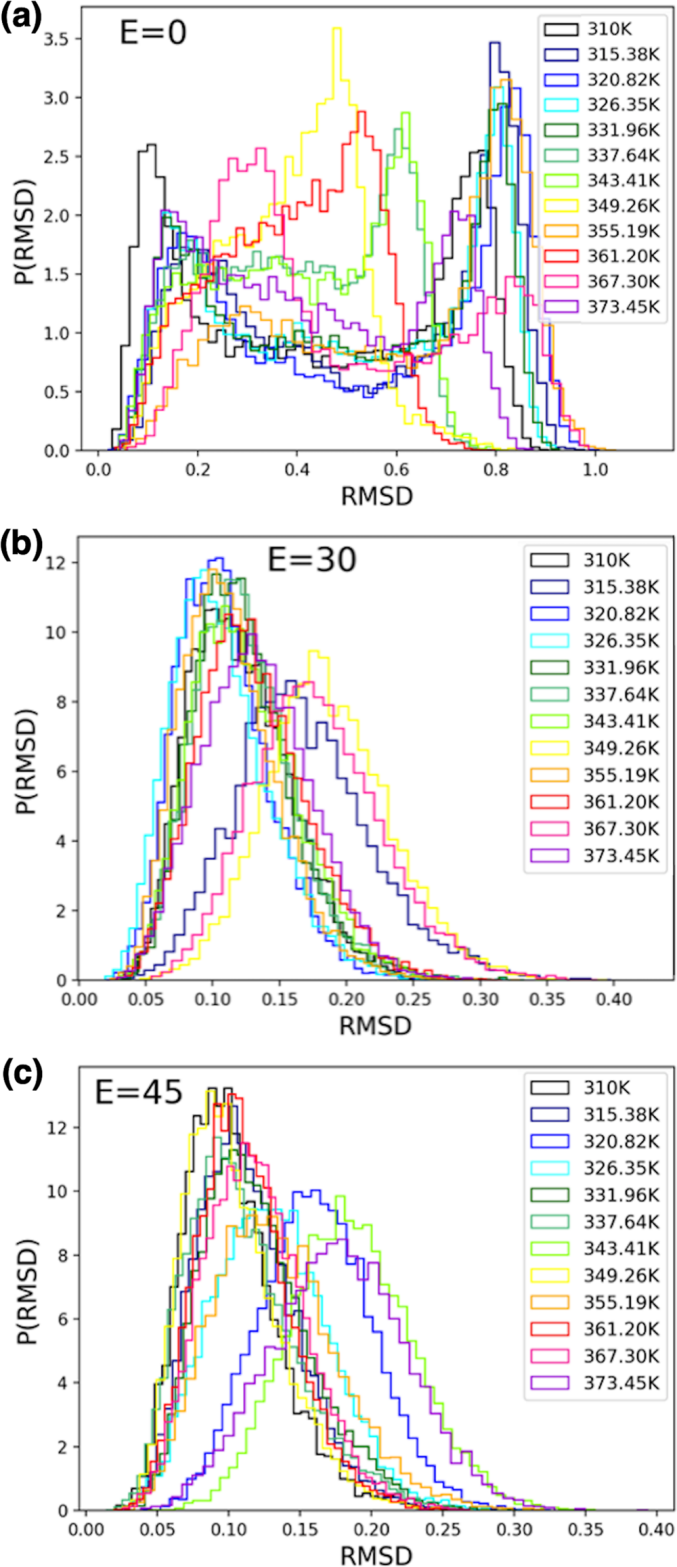
Distributions of RMSD values calculated for the heavy atoms of FF peptides for conformations from REMD simulations in the presence of external electric fields with intensities of (a) *E* = 0, (b) *E* = 30, and (c) *E* = 45 kcal/(mol Å e).

Figure 4 shows the distributions of root-mean-square deviation of atomic positions (RMSD) values calculated for the heavy atoms of FF peptides for conformations from REMD simulations in the presence of external electric fields with intensities in the three cases studied here and detailed in Table 1: *E* = 0, 30, and 45 kcal/(mol Å e), respectively. These distributions show clearly that the complexity of the conformational dynamics of the FF amyloid peptides is dramatically reduced in the presence of external fields, in agreement with earlier studies which, however, had significantly less sampling in simple MD simulations.^3^

**Figure 5:**
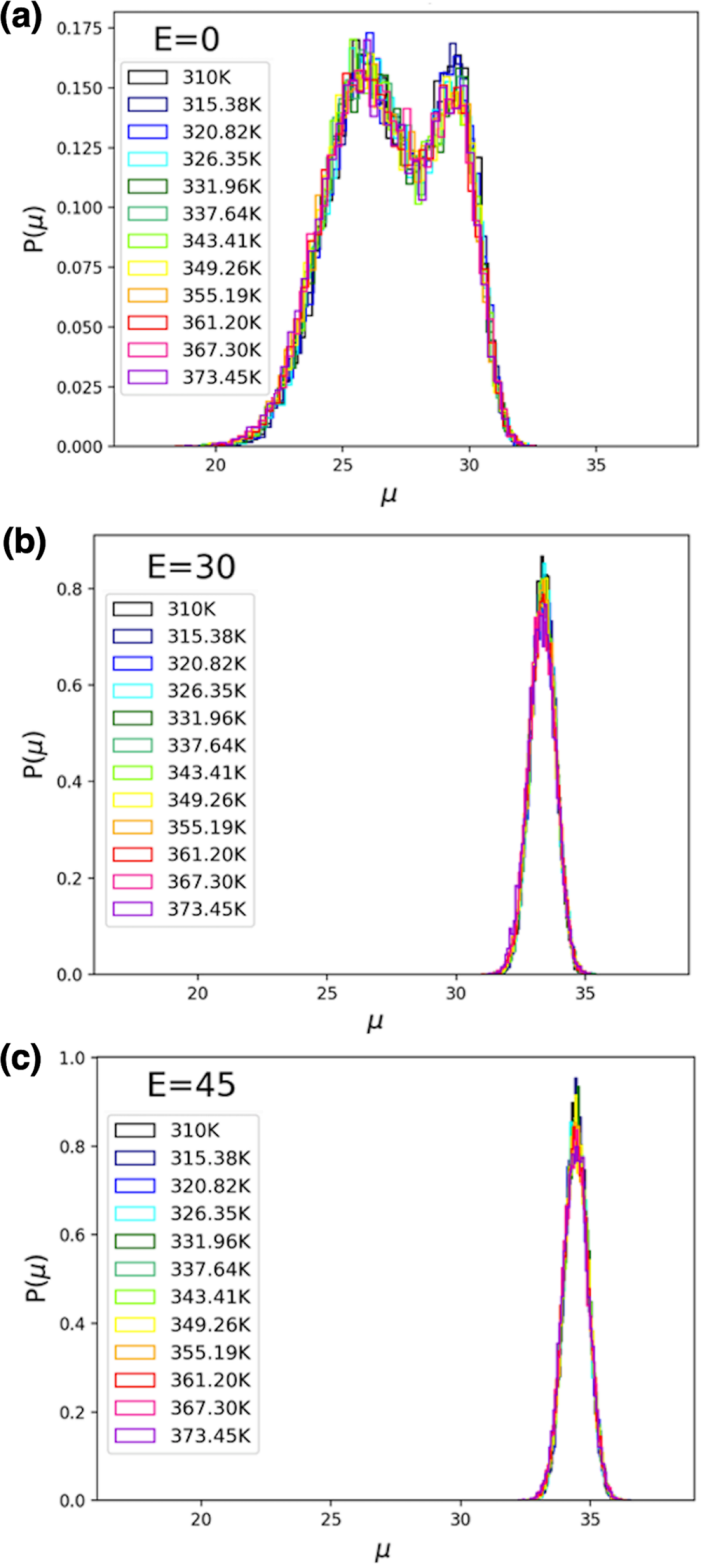
Distributions of the dipole moment magnitude (Debye units), calculated for FF peptides for conformations from REMD simulations in the presence of external electric fields with intensities of (a) *E* = 0, (b) *E* = 30, and (c) *E* = 45 kcal/(mol Å e).

In relation to the piezoelectric behavior of FF amyloid peptides, in Figure 5 are shown the distributions of the dipole moment magnitude (μ, Debye units), calculated for FF peptides for conformations from our three sets of REMD simulations in the presence of different external electric fields. In agreement with earlier observations, there is a noticeable effect on the magnitude of the dipole moment which increases systematically with larger *E* values, showing less complexity and fewer fluctuations at all temperatures, as the peptide adopts more extended conformations.

**Figure 6:**
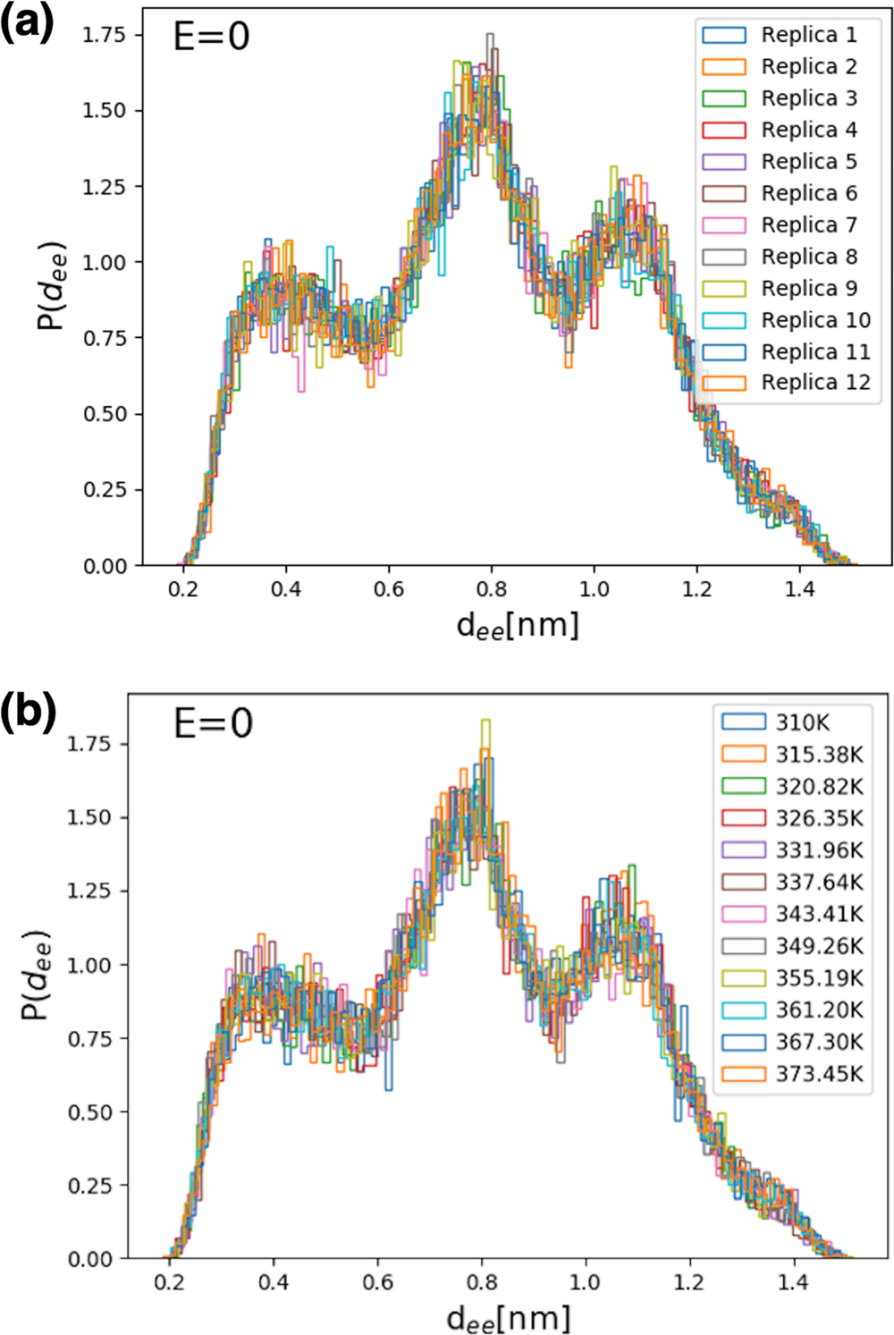
Replica exchange equilibrium distributions of side chain–side chain distances of FF amyloid peptides, with no external electric field applied, (a) for each replica (R-trajectories) and (b) at each temperature (T-trajectories) of the REMD simulation set.

However, while both RMSD and the dipole moment magnitude are useful collective variables utilized in MD analysis of FF peptides, Figures 4 and 5 also illustrate their intrinsic limitations in allowing us to identify and discuss the detailed dynamics. Thus, here we choose to focus on a different measure, the *d*_ee_ distances (i.e., distances between the C_*ζ*_ atoms at the ends of the two side chains, in Å, shown in Figure 1 in black), as a useful choice for our more detailed kinetic and thermodynamic analysis. Figure 6 shows REMD equilibrium distributions of *d*_ee_ values for FF amyloid peptides, in the case where no external electric field is applied, for each replica (R-trajectories, Figure 6a) and at each temperature (T-trajectories, Figure 6b) of the REMD trajectories. We note the clear presence of three conformational peaks.

**Figure 7:**
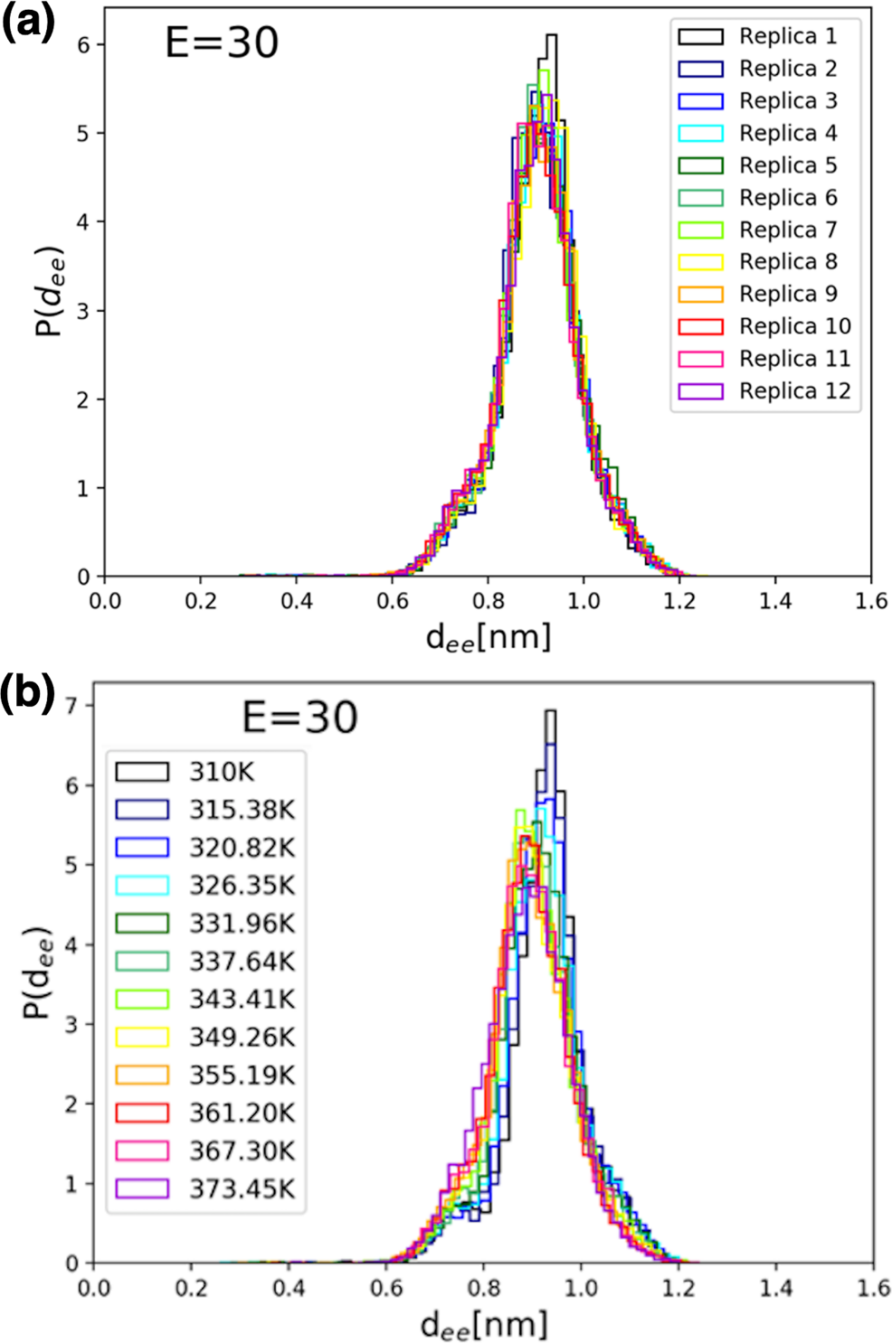
Distributions of side chain-to-side chain distances, *d*_ee_, for simulations with an applied electric field of 30 kcal/(mol Å e), (a) for each replica (R-trajectories) and (b) at each temperature (T-trajectories) of the REMD simulation set. Note that, at this field intensity, the conformational dynamics is restricted to an extended structure with a most probable *d*_ee_ value of ∼8.9 Å.

The corresponding distributions of side chain-to-side chain distances for simulations with an applied electric field of 30 kcal/(mol Å e) are shown in Figure 7 for each replica (R-trajectories, Figure 7a) and at each temperature (T-trajectories, Figure 7b) of the REMD simulation set. We note that, at a field intensity of 30 kcal/(mol Å e), the conformational dynamics is restricted to one extended structure with a most probable *d*_ee_ value of ∼8.9 Å.

**Figure 8:**
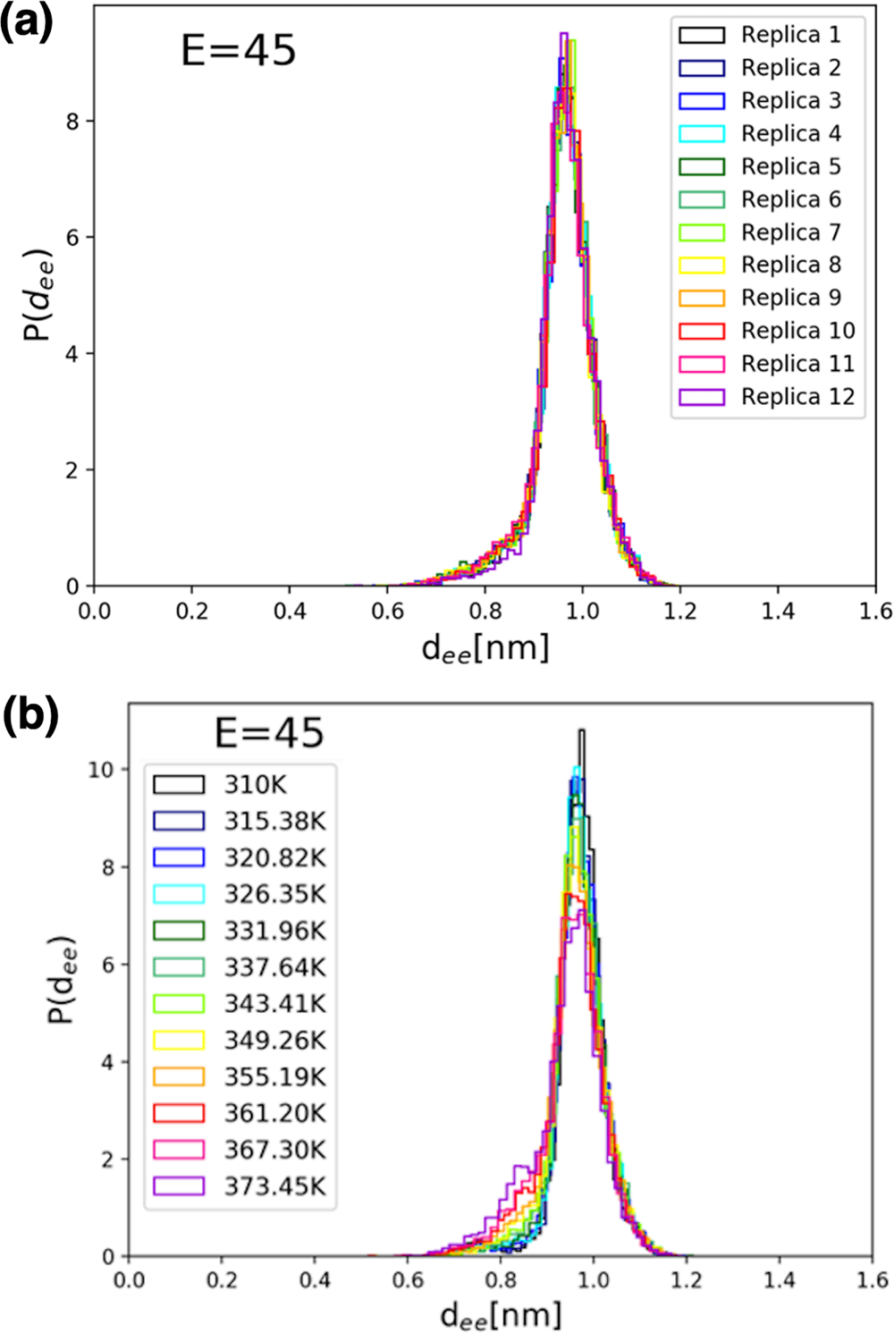
Distributions of *d*_ee_ values for simulations with an applied electric field of 45 kcal/(mol Å e), (a) for each replica (R-trajectories) and (b) at each temperature (T-trajectories) of the REMD simulation set. At this field intensity, the conformational dynamics is restricted further to a single extended structure with a most probable *d*_ee_ value of ∼10 Å.

Finally, in Figure 8 are shown the measured distributions of *d*_ee_ values for simulations with an applied electric field of 45 kcal/(mol Å e), for each replica (R-trajectories, Figure 8a) and, once again, at each temperature (T-trajectories, Figure 8b) of the REMD simulation set. At this field intensity, the conformational dynamics is restricted further to a single extended structure with a most probable *d*_ee_ value of ∼10 Å which, as expected, is a bit higher than in the previous case for an applied electric field of only 30 kcal/(mol Å e). As shown by data in Figures 5–8, our choice of electric field intensities, in agreement with earlier studies with less sampling,^3^ allows us to monitor the entire expected range of conformational changes that can occur when using a classical MD simulation force field. While the results are intrinsically limited by the classical nature of our MD simulations, they capture, nevertheless, the expected overall behavior of the FF system and allow us to study the response conformational dynamics electric field and temperature perturbations.

**Figure 9:**
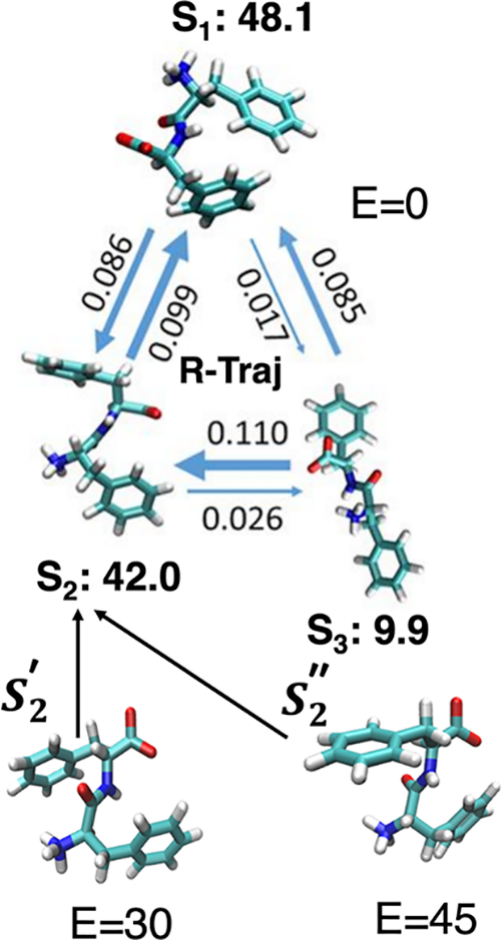
Representative conformations of FF amyloid peptides derived by kinetic analysis of REMD simulations at different electric fields. In the absence of electric fields, the FF peptide adopts three main Markovian conformational states, S_1_, S_2_, and S_3_ (top), with the corresponding state probabilities (in %) given next to each state label. The corresponding equilibrium transition rates between these states (blue arrows, see text) are also shown, as numbers. These REMD rates are for the data corresponding to all the replicas (all R-trajectories). Each arrow’s thickness is proportional to the magnitude of its corresponding transition rate. On the bottom are shown the representative conformations, S_2_′ and S_2_″, adopted in the presence of external electric fields with intensities of *E* = 30 and *E* = 45 kcal/(mol Å e), respectively.

The main results of our conformational and kinetic analysis are summarized in Figure 9. Here, the temperature-dependent Markov kinetic network is illustrated, estimated from our new REMD simulation trajectories for FF peptides in the absence (top) and the presence of representative electric field intensities. Figure 9 shows the relative transition probabilities (blue arrows) between the three major conformational Markovian states (denoted as S_1_, S_2_, and S_3_) and their corresponding probabilities of occurrence (or state populations in percentages). Note that, in the absence of electric fields (Figure 9, top), the FF peptide adopts three different main Markovian conformational states: S_1_, S_2_, and S_3_. In Figure 9, the corresponding equilibrium transition rates between these states (blue arrows, see text) are shown as numbers. The REMD transition rates were extracted for the data corresponding to transitions occurring in all trajectories, cumulated for all the replicas (all R-trajectories). As shown in our earlier work on analyzing and extracting kinetic information from REMD data from different atomistic systems (e.g., pentaalanine^49^ and NNQQ^28,32^ peptides), while the data from all the R-trajectories correspond to dynamics at an intermediate temperature that is not exactly defined, they are nevertheless representative for the entire set of REMD replicas, at all temperatures. Moreover, the propagators for transitions along R-trajectories can be calculated analytically as weighted geometric means of propagator values extracted for the corresponding transitions in T-trajectories.^28^ In Figure 9, each arrow’s thickness is proportional to the magnitude of its corresponding transition rate. On the bottom are illustrated the representative FF conformations, denoted here as S_2_′ and S_2_″, adopted by the peptide in the presence of external electric fields with intensities of 30 and 45 kcal/(mol Å e), respectively. As illustrated in Figure 9 (and as suggested by the notation), our REMD simulations show that the S_2_′ and S_2_″ conformations induced by the external electric field, at different field magnitudes, are part of the same conformational ensemble as the S_2_ conformations adopted intrinsically by the FF peptide even in the absence of an externally applied electric field, but with a probability of only ∼42%. The S_2_-type of molecular conformations, shown in Figure 9, results from the peptide backbone stretching effect due to the presence of the external field and results in a more direct exposure of the hydrophobic aromatic rings of the phenyl side chains to peptide–peptide interactions facilitating FF aggregation. The interplay between increased backbone dipolar moments and stronger side chain–side chain interactions could be particularly important in understanding the dependence of FF–peptide aggregation propensities on physical parameters such as temperature and external electric fields.

## Conclusions

In summary, we show that replica exchange molecular dynamics (REMD) trajectories of explicitly solvated FF peptides can be used to probe in detail the interplay between temperature and electric field effects on the detailed thermodynamic and kinetic properties of the conformational dynamics of FF peptides in the presence of explicit water molecules.^3,4^ While their well-documented piezoelectric properties allow FF molecules and their aggregates (e.g., FF nanotubes) to be aligned in a controlled way by the application of external electric fields, the detailed responses of individual peptides to both temperature and electric fields are not fully understood. Here, we show that the thermodynamics and kinetics of the ensemble of conformations adopted by amyloid FF peptides solvated in explicit water molecules—an environment relevant to biomedical applications—can be analyzed in detail by using REMD to enhance sampling, while simultaneously applying external electric fields and probing temperature ranges relevant to earlier studies.^3,9,10,14,15^

Methodologically important, our simulations highlight and overcome possible artifacts that may occur during the setup of REMD simulations of explicitly solvated peptides in the presence of external electric fields, a problem particularly important in the case of short peptides such as FF. The presence of the external fields could overstabilize certain conformational states in one or more REMD replicas, leading to distortions of the underlying potential energy distributions observed at each temperature. This cause is different from REMD artifacts reported and documented by earlier studies, which were due to modified underlying energy distributions caused, for example, by the use of weak-coupling thermostats.^47,48^ In our case, we show that the resulting artifacts can be overcome by correcting the REMD initial conditions to include the lower-energy conformations induced by the external field. This is illustrated by the initial energy distributions shown in Figure 2 and the corrected ones from Figure 3. Such corrections could also be important in other replica-exchange simulations (e.g., using methods such as REST2^50,51^) that enable the use of a broader range of temperatures in atomistic MD studies of amyloid peptide aggregation.^52^

Subsequently, we show that the corrected and converged REMD data can be analyzed using a Markovian description of conformational states and show that a rather complex, 3-state, temperature-dependent conformational dynamics in the absence of electric fields collapses to only one of these states in the presence of the electric fields. As illustrated in Figure 9, we can study and analyze the detailed interplay between the temperature and electric field on the thermodynamic and kinetic properties of solvated FF peptides. In particular, we identify and characterize the ensemble of S_2_-type molecular conformations, illustrated in Figure 9, which are expected to play a particularly important role in understanding the dependence of FF–peptide aggregation propensities on physical parameters such as temperature and external electric fields. The mechanistic details behind the temperature- and electric-field-dependent thermodynamic and kinetic properties of small FF amyloid peptides can be useful in understanding and devising new methods to control their aggregation-prone biophysical properties and, possibly, to modulate the structural and biophysical properties of FF molecular nanostructures.

## Abbreviations

FF: diphenylalanine
DTC: direct transition counting
MD: molecular dynamics
MLPB: maximum likelihood propagator-based
REMD: replica-exchange molecular dynamics
RMSD: root-mean-square deviation
TBA: transition-based assignment.
